# Optoelectronic intelligence

**DOI:** 10.1063/5.0040567

**Published:** 2021-04

**Authors:** Jeffrey M. Shainline

**Affiliations:** National Institute of Standards and Technology, Boulder, Colorado 80305, USA

## Abstract

General intelligence involves the integration of many sources of information into a coherent, adaptive model of the world. To design and construct hardware for general intelligence, we must consider principles of both neuroscience and very-large-scale integration. For large neural systems capable of general intelligence, the attributes of photonics for communication and electronics for computation are complementary and interdependent. Using light for communication enables high fan-out as well as low-latency signaling across large systems with no traffic-dependent bottlenecks. For computation, the inherent nonlinearities, high speed, and low power consumption of Josephson circuits are conducive to complex neural functions. Operation at 4 K enables the use of single-photon detectors and silicon light sources, two features that lead to efficiency and economical scalability. Here, I sketch a concept for optoelectronic hardware, beginning with synaptic circuits, continuing through wafer-scale integration, and extending to systems interconnected with fiber-optic tracts, potentially at the scale of the human brain and beyond.

## I. INTRODUCTION

General intelligence is the ability to assimilate knowledge across content categories and to use that information to form a coherent representation of the world. The brain accomplishes general intelligence through many specialized processors performing unique, complex computations.^1,2^ The information generated by these processors is communicated throughout the network via dedicated connections spanning local, regional, and global scales.^3^ On the microscale, synapses, dendrites, and neurons are specialized processors comprising the gray matter computational infrastructure of the brain.^4^ On the meso-scale, cortical minicolumns of 100 neurons act as specialized processors,^5^ and on the macro-scale, brain regions play that role.^6^ Information is communicated between these modules via axonal fibers that comprise the white matter communication infrastructure of the brain. On short time scales, information processing occurs in synapses,^7^ dendrites,^8^ and within single neurons.^9^ On longer time scales, the information generated by minicolumns is communicated across wider regions of the network so that the knowledge of specialized processors can combine in a comprehensive interpretation of a subject.^10^ The utilization of many specialized processors combining their shared computational resources across many scales of space and time enables the brain to achieve general intelligence.^1,2^

Computation and communication are the complementary pillars of neural systems. Hardware for artificial general intelligence (AGI) will achieve the highest performance if complex, local processors can pool the information from their specialized computations through global communication. Electrons excel at computation, while light is excellent for communication. In silicon hardware, monolithic optical links between a processor and memory have been demonstrated.^11^ These devices were fabricated in a 45-nm CMOS node with no in-line process changes, and off-chip light sources were utilized. Such work is driven by the desire for increased communication bandwidth in multicore architectures. These architectures continue to expand into on-chip networks, in some cases resulting in highly distributed, brain-inspired systems implemented with CMOS electronics.^12–17^ As computing grows more distributed, communication becomes a bottleneck. A primary challenge affecting further chip-scale electronic-photonic integration is the difficulty of achieving a light source on silicon that is robust, efficient, and economical.^18,19^ Lessons learned from very-large-scale integration (VLSI) inform us that economical fabrication of integrated circuits comprising simple components is necessary for scaling. In this regard, difficulties associated with integrated light sources are the most significant impediment to optoelectronic VLSI.

It is the perspective of our group at NIST that hardware incorporating light for communication between electronic computational elements combined in an architecture of networked optoelectronic spiking neurons may provide potential for AGI at the scale of the human brain. Spiking neurons are circuits that integrate signals over time and produce pulses when a threshold is reached. The spiking neurons discussed here are optoelectronic in that the pulses communicated from neurons to synapses consist of photons, while the computations performed within the neurons utilize electronics. Each neuron contains a light source, which is driven electrically upon reaching threshold. Each synapse contains a detector, which converts the optical signal to an electrical current or voltage upon receiving a photonic synapse event. Each neuron is a distinct entity, and no hardware components are multiplexed to represent the operations of separate neurons at different times. While much of present-day computing infrastructure has evolved to implement a von Neumann architecture performing sequential operations in the model of a Turing machine, the functioning of neural systems departs considerably from this model. Light has even more to offer in a neural computing context, because communication across scales is indispensable. Further, the spiking behavior of Josephson junctions (JJs) combined with the efficiency of single-photon detectors (SPDs) make a compelling case for optical integration with superconducting electronics.^20,21^ Such a choice necessitates low-temperature operation near 4 K. At this temperature, silicon light sources become available,^22^ indicating that a major impediment to optoelectronic VLSI many not be present in the superconducting domain. This article summarizes the reasoning behind the assertion that superconducting optoelectronic systems have unique potential to achieve general intelligence when considered from the perspectives of cognitive science and VLSI.

The unique cognitive capabilities of humans derive in part from the scale of the brain, including the number of neurons and the complexity of the communication network. While there is much to be gained from artificial intelligence (AI) hardware at smaller scales, this article considers technological pathways to large cognitive systems, with tens to hundreds of billions of neurons, and communication infrastructure of commensurate complexity. Such technology will likely require many interconnected wafers, each packed densely with integrated circuits. We may refer to this field of research as “neuromorphic supercomputing.” The effort is in some ways more akin to the construction of a fusion reactor or particle accelerator than a microchip, and potentially offering a similar scale of societal benefit in the form of an experimental test bed enabling the elucidation of the mechanisms of cognition and the exploration of the physical limits of intelligence.

## II. NEUROSCIENCE AS A GUIDE

To guide the design of hardware for AGI, we must consider insights from neuroscience regarding how neural systems integrate information across space and time to accomplish cognition.^3,10,23,24^ A brief summary is provided here, highlighting aspects most pertinent to the design of hardware for cognition.

In the temporal domain oscillations and synchronization structure the activity of populations of neurons.^10^ The spiking activity of neurons is observed to comprise nested oscillations across a range of frequencies.^25^ On the fastest time scales of the brain, local clusters of neurons engage in transient dynamical activity induced by the present stimulus. These patterns of activity are referred to as gamma oscillations (80 Hz), and activity in this band is modulated by lower-frequency oscillations^26,27^ resulting from the combined activity of neurons across larger regions of the network.^28^ These slower, broader patterns are referred to as theta oscillations (6 Hz), and neuronal communication across a network depends upon information present in gamma activity being structured into more complex syntax by dynamics on theta timescales.^29,30^ This rich structuring of information in time is enabled by the spiking behavior of neurons. Computation and communication based on spikes facilitate a diversity of information coding schemes with resilience to noise while maintaining high energy efficiency due to sparse activity.

In the spatial domain, a feature of neural systems that will recur in the present discussion is their modular, hierarchical construction.^3,23,24,31^ Neural systems are modular in that they are comprised of local regions of densely interconnected structures with sparser connectivity between such regions. Neural systems are hierarchical in that this pattern repeats across spatial scales in a fractal manner: minicolumns aggregate into columns, columns into complexes, etc. This fractal property is necessary to enable networks to scale arbitrarily, with dynamics constrained only by the physical hardware and spatial extent of the system rather than by the ability to communicate across the network.^32^ Communication between distant modules is enabled by power-law scaling: the number of connections being sent to distant modules does not decay exponentially, but rather follows a power law.^33,34^ The non-vanishing tail of long-range connections enables distant modules to quickly become correlated. In constructing hardware for artificial intelligence, it is imperative to enable rapid communication without traffic-dependent bottlenecks. Modules must be able to quickly engage in gamma activity, while signals from many interconnected modules at multiple levels of hierarchy must be able to simultaneously transmit across the complex network. The specific time scales defining behavior analogous to gamma and theta oscillations will be determined by the underlying computational devices.

**FIG. 1.**
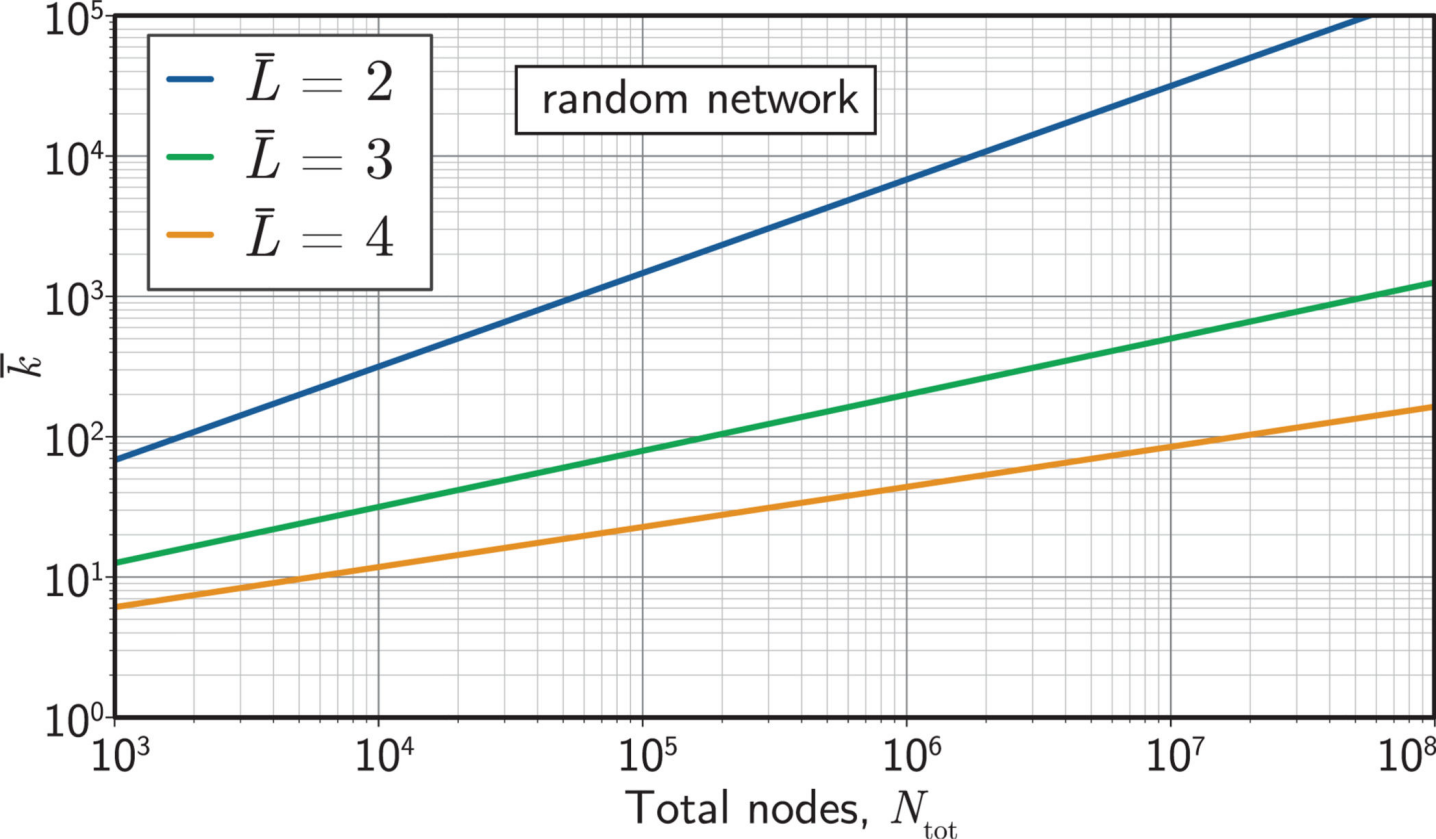
The average number of connections per node ($\bar{k}$) required to maintain a given average path length ($\bar{L}$) across a random network as a function of the total number of nodes in the system (*N*_tot_).

In the form of gamma activity, clusters of neurons represent specific content, and the information from these clusters must be shared with other regions of the network to form a multifaceted representation of a stimulus. This computation and communication is facilitated by networks with a high clustering coefficient yet also an average path length nearly as short as a random graph.^38^ In the language of network theory, if node *a* is connected to node *b*, and *b* is connected to *c*, then clustering quantifies the probability that *a* will be connected to *c*. Path length quantifies the number of intermediate nodes that must be traversed to get from one node to another along the network connections. The average path length is determined by calculating this quantity over all pairs of nodes in the network, and taking the mean. A network with high clustering and low average path length is referred to as a “small-world network.”^39^ Small-world networks are ubiquitous throughout the brain^3^ and require long-range connections. In a random network, near and distant connections are equally probable, so the average path length across the network achieves a lower limit on path length for a given number of edges connecting a given number of nodes. Figure 1 shows the number of edges required per node to achieve a given average path length as a function of the number of nodes in the random network. For a modest network with one million nodes, each node must make several thousand connections to maintain a path length of two. For the case of a network with 10^8^ nodes, each node must make over one hundred thousand connections. This is similar to the hippocampus in the human brain, with nearly 10^8^ neurons, some with 50 000 or more nearly random synaptic connections.^10^ Maintaining a short path length across the network is critical for information integration, and is an important motivator to use light for communication.

At the device level, dynamical behaviors thought to be necessary for attention, cognition, and learning, such as cross-frequency coupling^10^ and synaptic plasticity,^40–42^ require complex capabilities. Dynamical synapses, dendrites, and neurons allow one structural network to realize myriad functional networks adapting on multiple time scales. While light is excellent for communication, electrical circuits are better equipped to perform these nonlinear, dynamical functions. For communication and computation, neural information processing will benefit immensely from optoelectronic integration.

**FIG. 2.**
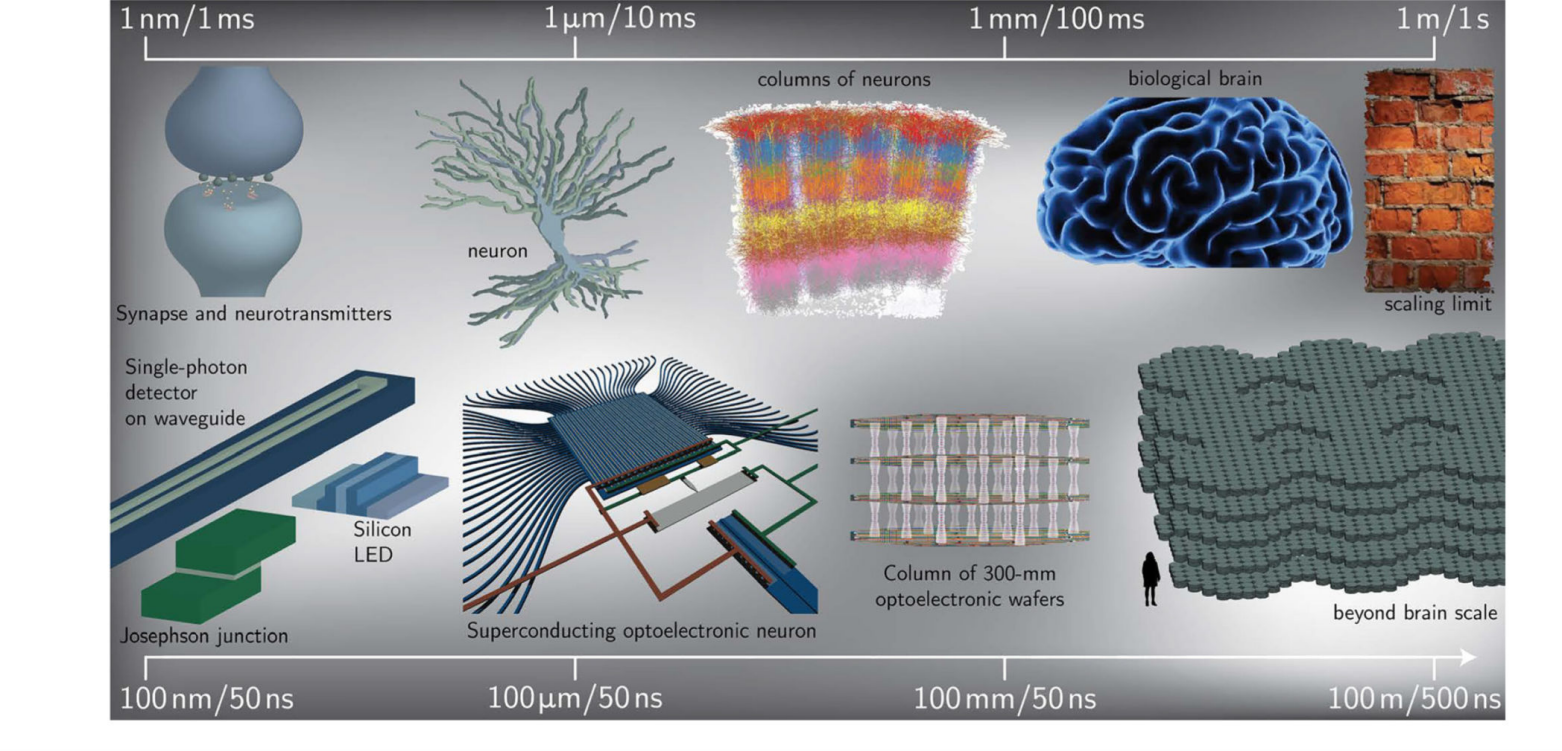
Structure across scales. Biological systems have functional components from the nanometer scale (neurotransmitters, axonal pores) up to the full brain (0.3 m linear dimension for full human cerebral cortex^35^). Speeds are limited by chemical diffusion and signal propagation along axons, which may ultimately limit the size of biological neural systems.^10,36^ The time constants associated with chemical diffusion and membrane charging/discharging span the range from 1 ms to 100 ms^4,37^ and dictate the speeds at which information processing occurs. The wall at the right indicates the communication-limited spatial-scaling barrier. Optoelectronic devices rarely have components with critical dimension smaller than 100 nm, and optoelectronic neurons are likely to be on the 100-*μ*m scale, with dendritic arbor extending for millimeters and axonal arbor in some cases spanning the system. The time constants of these components can be engineered in hardware across a very broad range with high accuracy through circuit parameters, enabling rapid processing as well as long-term signal storage. Optical communication enables optoelectronic systems to extend far beyond the limits imposed by the slow conduction velocity of axons.

Figure 2 charts the structures present on various scales for biological and optoelectronic hardware. The human brain has features spanning roughly eight orders of magnitude in size, from a nanometer to a tenth of a meter. Across time, activity ranges from the 1 ms timescale of neurotransmitter diffusion across a synapse, through the 200 ms timescale of brain-wide theta oscillations, up to the memory retention time of the organism. The speeds of devices and communication in the brain are limited by the chemical and ionic nature of various operations. The maximum size of the brain may be limited by the slow conduction velocity of ionic signals along axons. If the brain were larger, signals would not have time to propagate between different regions during the period of theta oscillations, and system-wide information integration could not be efficiently achieved.^10,36^

Light and electronics together can enable communication and computation across spatial and temporal scales. We have proposed a specific approach we see as most conducive to large-scale implementation for AGI.^20,21,36,43^ The approach combines waveguide-integrated light sources and SPDs for communication^20,22^ with Josephson circuits for synaptic, dendritic, and neuronal computation.^21,43^ As illustrated in Fig. 2, these optoelectronic networks will have features as small as 100 nm and potentially extend up to kilometers. Neuronal interspike intervals can be as short as 50 ns, while synaptic and dendritic processing occurs on the 50 ps timescale of Josephson junctions. Time constants can be chosen across many orders of magnitude, enabling information processing and memory across time scales. Figure 2 is intended to emphasize that if communication barriers can be removed, neural systems of extraordinary scale can be achieved.

**FIG. 3.**
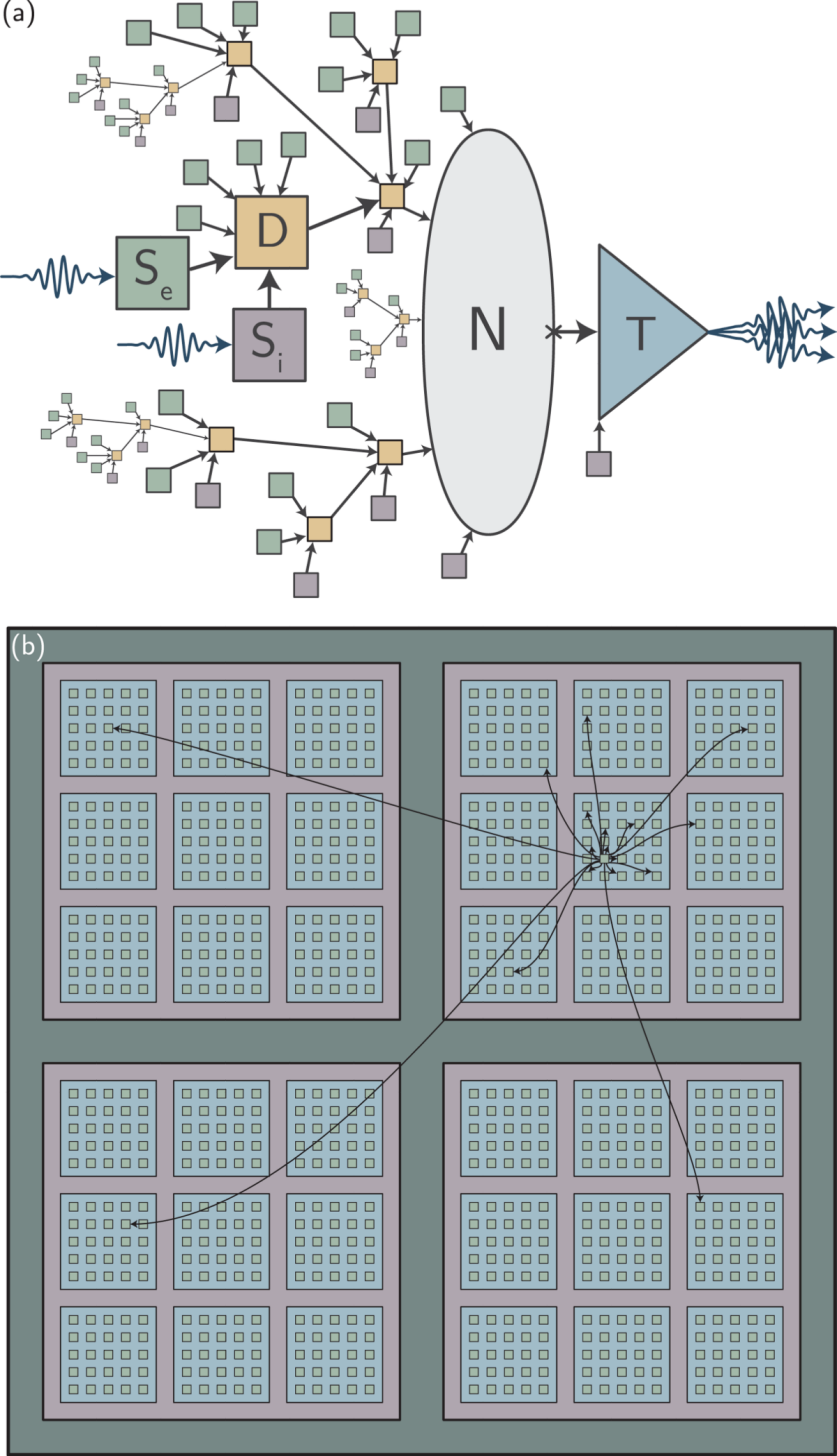
Block diagrams. (a) Optoelectronic neuron. Electrical connections are shown as straight, black arrows, and photons are shown as wavy, blue arrows. (b) Modular, hierarchical network construction. Here black arrows are photonic connections. Part (a) adapted from J. M. Shainline, IEEE J. Sel. Top. Quantum Electron. 26, 1 (2020). Copyright 2020 Author(s), licensed under a Creative Commons Attribution (CC BY) license.^43^ Part (b) adapted from Shainline *et al.*, J. Appl. Phys. 126, 044902 (2019). Copyright 2019 Author(s), licensed under a Creative Commons Attribution (CC BY) license.^36^

Schematic illustrations of the neurons and modular networks under consideration are presented in Fig. 3. A neuron with a complex dendritic tree is shown in Fig. 3(a). The neuron comprises excitatory (S_e_) and inhibitory (S_i_) synapses feeding into dendrites (D) and the neuron cell body (N). Upon reaching threshold, the transmitter (T) produces a pulse of light that fans out across a network of waveguides (not shown). Modular hierarchical construction is depicted in Fig. 3(b). The smallest blocks represent neurons, and their connections predominantly reside within their local module (blue). Yet important connections are made at all levels of hierarchy (red and dark green). Hardware for AGI must employ modular, hierarchical networks of complex neurons with rich dynamics that adapt on multiple timescales.

## iii. SUPERCONDUCTING OPTOELECTRONIC SYNAPSES, DENDRITES, AND NEURONS

**FIG. 4.**
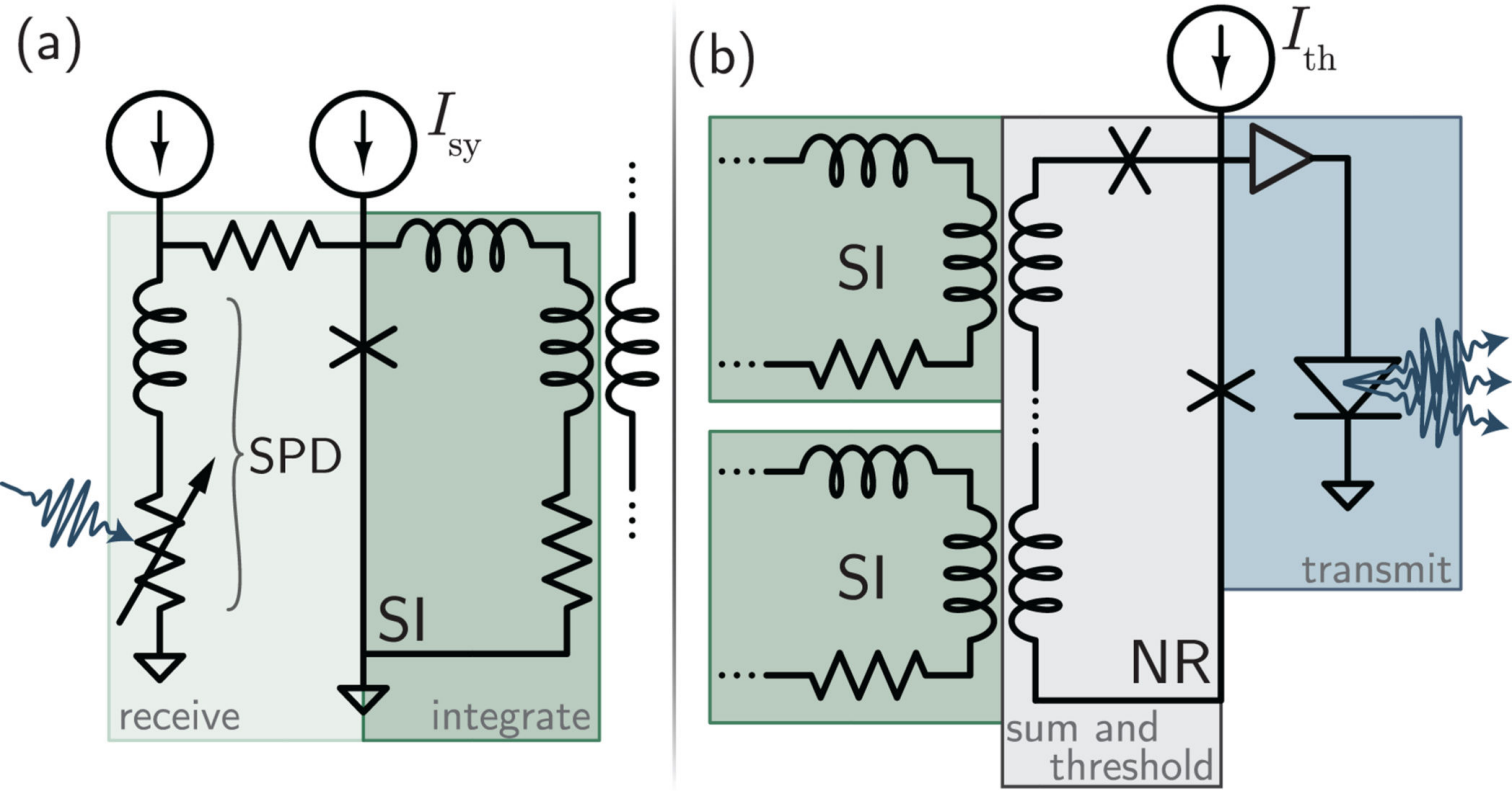
Circuit diagrams. (a) Superconducting optoelectronic synapse combining a single-photon detector (SPD) with a Josephson junction and a flux-storage loop, referred to as the synaptic integration (SI) loop. The synaptic bias current (*I*_sy_) can dynamically adapt the synaptic weight. (b) Neuron cell body performing summation of the signals from many synapses as well as thresholding. Here the neuronal receiving (NR) loop is shown collecting inputs from two SI loops, but scaling to thousands of input connections appears possible. Upon reaching threshold, the transmitter circuit (amplifier^47^ and LED^22^) produce a pulse of light that communicates photons to downstream synapses. The neuronal threshold current (*I*_th_) can dynamically adapt the neuronal threshold.

Having chosen to communicate synaptic events with light, the quantum limit is a single photon per synaptic connection. We have designed a synapse [Fig. 4(a), Refs. 21, 36, and 43] that detects a single near-infrared photon and requires no power to retain the synaptic state, a feature enabled by the dissipationless nature of superconductors. The synapse utilizes a superconducting-nanowire SPD, which is simply a current-biased strip of superconducting wire.^44^ To achieve the desired synaptic operation, an SPD is combined in circuits with Josephson junctions (JJs) and superconducting loops to achieve the functions needed for neural information processing. In optoelectronic synapses of this design, the current bias across a single JJ establishes the synaptic weight [*I*_sy_ in Fig. 4(a)]. This current bias can be dynamically modified through various photonic and electronic means based on control signals or network activity.

The signals from many synapses can be combined through transformers coupled to dendrites or neurons [Fig. 4(b)]. Neurons constructed in this manner are highly modular in that synapses, dendrites, and the neuron cell body itself are all based on the same core circuit, comprising a superconducting quantum interference device (SQUID) embedded in a flux-storage loop. SQUIDs are perhaps the most ubiquitous of all superconducting circuits,^45,46^ often used as sensors due to their extraordinary sensitivity to magnetic flux and low-noise operation. These properties make SQUIDs ideal circuits for dendrites and neurons to perceive and respond to minute changes in analog signal levels. Dendritic and neuronal nonlinearities are a natural consequence of the JJ critical current, and can be shaped through the choice of circuit parameters, such as loop inductances and resistances, as well as dynamically with adaptive bias currents. Due to the prominent role of superconducting current storage loops, we refer to these as loop neurons. We refer to networks of loop neurons as superconducting optoelectronic networks (SOENs). In the operation of loop neurons, a single photon triggers a synaptic event, and spike-timing-dependent plasticity is induced by two photons—one from each neuron associated with the synapse.

In addition to the choice of SPDs as the detectors in the system, we must also select a light source, which must be fabricated across wafers by the millions. Because our choice of detectors dictates cryogenic operation, silicon light sources are an option. The light sources we have in mind are silicon LEDs,^22^ employing luminescence from defect-based dipole emitters. From the perspective of VLSI, achievement of a silicon light source as simple as a transistor would be the greatest contribution to the success of this technology. If cryogenic operation enables both SPDs and silicon light sources, it will be worth the added infrastructure for cooling. We further justify this decision in Sec. IV.

**FIG. 5.**
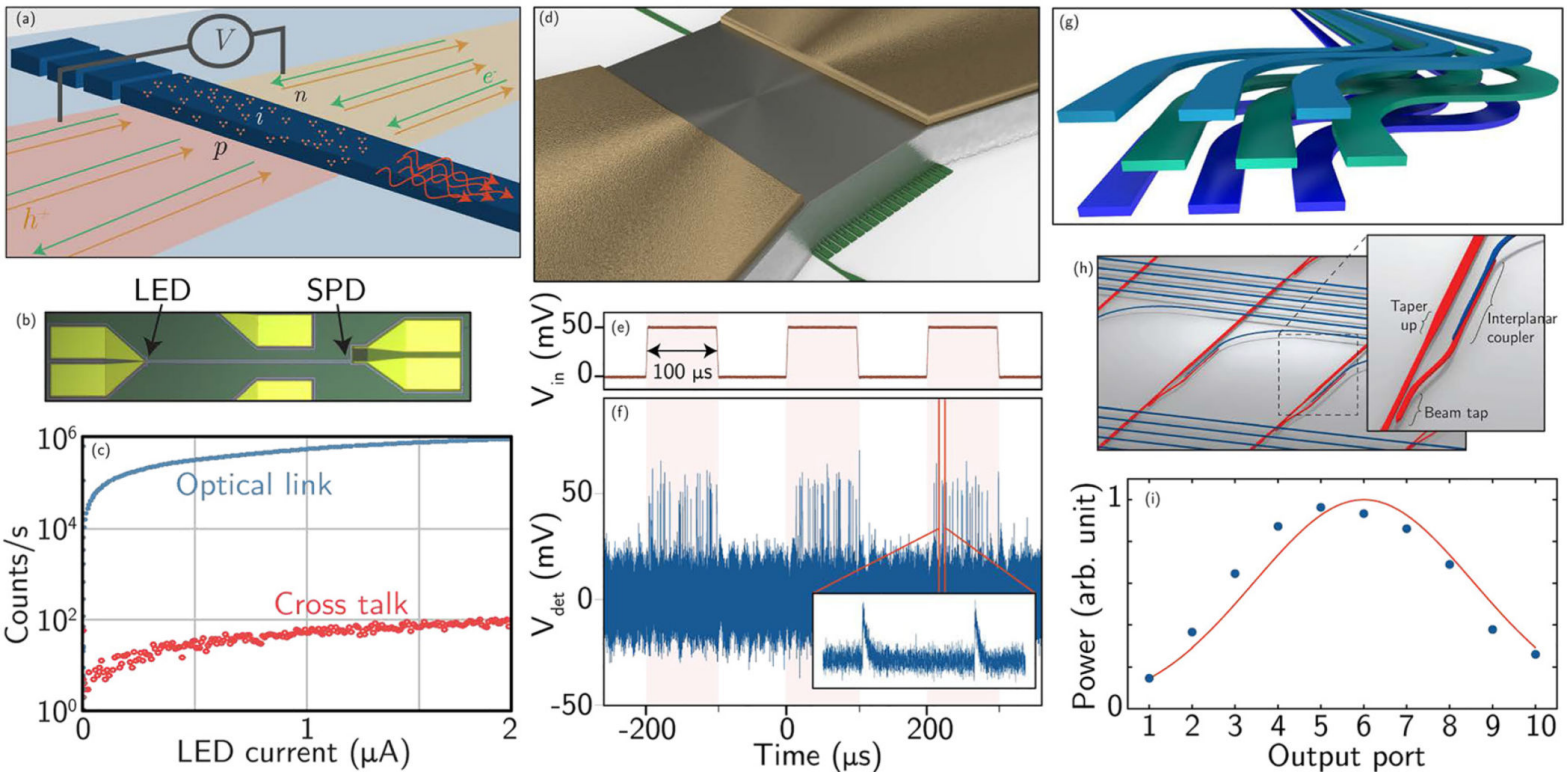
Experimental progress toward superconducting optoelectronic networks. (a) Schematic of waveguide-integrated silicon LED. (b) Microscope image of a silicon LED waveguide-coupled to a superconducting-nanowire detector. (c) Experimental data showing that light is coupled through the waveguide, while crosstalk to an adjacent detector on the chip is suppressed by 40 dB. (a)–(c) Adapted from Buckley *et al.*, Appl. Phys. Lett. 111, 141101 (2017). Copyright 2017 Author(s), licensed under a Creative Commons Attribution (CC BY) license.^22^ (d) Schematic of the superconducting thin-film amplifier. (e) and (f) The resistive switch driving the LED. (e) Square pulses are driven into the switch gate. (f) When the switch is driven, light is produced from the LED and detected by the SPD. (d)–(f) Adapted from McCaughan *et al.*, Nat. Electron. 2, 451 (2019). Copyright 2019 Author(s), licensed under a Creative Commons Attribution (CC BY) license.^47^ (g) Schematic of multi-planar integrated waveguides for dense routing. Adapted from Chiles *et al.*, APL Photonics 2, 116101 (2017). Copyright 2017 Author(s), licensed under a Creative Commons Attribution (CC BY) license.^48^ (h) Schematic of feed-forward network implemented with two planes of waveguides. (i) Data from an experimental demonstration of routing between nodes of a two-layer feed-forward network with all-to-all connectivity. (h) and (i) Adapted from Chiles *et al.*, APL Photonics3, 106101 (2018). Copyright 2018 Author(s), licensed under a Creative Commons Attribution (CC BY) license.^49^

To achieve complex neural circuits, we aim for monolithic integration of light sources, detectors, and superconducting circuit elements. Our group’s experimental progress toward this end is summarized in Fig. 5. An important milestone was the demonstration of an all-silicon monolithic optical link. We measured waveguide coupling of light from micrometer-scale, all-silicon LEDs to integrated, silicon-based SPDs on a photonic chip [Figs. 5(a)–5(c), Ref. 22]. Further progress on scalability and characterization of waveguide-integrated SPDs for use in the synapses under consideration was also presented in Ref. 50. The performance achieved in the first iteration of these optical links was not yet adequate. The observed efficiency was 5 × 10^−7^, while 10^−3^ or higher is desirable for large systems.^36^ Yet the simplicity of both the source and detector made the fabrication and demonstration of a monolithic optical link far easier than if room-temperature operation were required. Subsequent work improved the brightness of the sources by two orders of magnitude through optimized fabrication procedures.^51^ Additional gains may result from optimization of the diode structure used for electrical injection of carriers into the waveguide where electron-hole recombination at emissive centers produces waveguide-coupled luminescence. Elimination of etched surfaces and proper passivation in the active region may significantly reduce non-radiative recombination. Improvements to the optical structure may increase coupling efficiency from the emitters to the waveguide mode. For this application, the light sources are only required to produce incoherent pulses of 10 000 photons (1 fJ) at 20 MHz when operating at 4 K. Modest advances could enable silicon light sources to meet these specifications.

We have also demonstrated superconducting amplifiers capable of generating the voltage required to produce light from these sources [Figs. 5(d)–5(f), Ref. 47]. Generating more than a millivolt with superconducting circuits is difficult, but the thin-film, micrometer-scale cryotron demonstrated in Ref. 47 leverages the extreme nonlinearity of the superconducting phase transition to rapidly generate high impedance and voltage with low energy, thus driving a semiconductor light source during each neuronal firing event. In Ref. 47, we demonstrated the use of these amplifiers to drive the LED-SPD link of Ref. 22. Fabrication of these devices appears compatible with silicon microelectronic manufacturing, provided the high-temperature steps required for dopant activation and contact annealing required for semiconductor devices are performed prior to the deposition of superconducting thin films.

Following a neuronal spike, the light produced by an amplifier driving an LED fans out across a network of micrometer-scale dielectric waveguides terminating on the superconducting detectors at each synaptic connection. We have demonstrated multiple vertically integrated planes of these waveguides [Figs. 5(g)–5(i), Refs. 48 and 49], and used them to implement the architecture of a feed-forward neural network with two layers of 10 neurons per layer and all-to-all connectivity.^49^

## IV. THE LANDSCAPE OF RESEARCH IN PHOTONIC AND SUPERCONDUCTING NEURAL SYSTEMS

This approach to neural computing resides at the confluence of semiconductors, superconductors, and photonics, and should be contextualized with other work in these fields. It is clarifying to acknowledge that nearly all efforts to use photonics or superconducting electronics for neural systems are focused on the entirely reasonable goal of doing useful computations with hardware that is available right now. These efforts are valuable and promising for their own ends without seeking brain-scale cognition. Comments here contrasting SOEN hardware with other current efforts are not criticisms of any work in the field, but rather explanation of the reasoning behind SOENs for large cognitive systems. A short summary of other photonic and electronic efforts is provided here, and comprehensive reviews of emerging neural hardware can be found in recent literature.^52–54^

### A. Semiconductor electronic neural systems

Given the extraordinary success of CMOS electronics, utilization of that hardware platform is the clear place to begin a search for artificial neural circuits. The history of exactly this pursuit is rich,^55–57^ accomplished,^58–62^ and exciting advances lie ahead.^63^ So why advocate for an alternative? To further explain why we place optical communication at the center of hardware development, I briefly summarize the physical limitations of electrical interconnection networks.^64^ It is impracticable in silicon electronics for a single device to source current to many other devices. A shared communication network must be employed. Switched media networks are used for this purpose. Each device must then only communicate to the nearest switch in the network. Because the communication infrastructure is shared, devices must request and wait for access to the switch network to transmit messages. This approach to communication leverages the speed of electronic circuits to compensate for the challenge of direct communication. Limitations are reached when many devices must communicate with many other devices simultaneously. While neural activity is generally sparse, during high activity, such as coordinated gamma bursting at the peak of a theta oscillation (Sec. II), many neurons must communicate simultaneously across the network. Due to the traffic-dependent bottleneck of shared interconnection infrastructure, as more neurons are added to the network, the average rate of neuronal firing events must decrease, and nested oscillations must shift to lower frequencies due to delays. Activity is limited to frequencies much slower than the brain in systems much smaller than the brain. Integration of information across the network is limited by the communication infrastructure.

### B. Optical neural systems

One means to alleviate communication limitations is through the use of optics. The field of photonic neural systems began^65,66^ with an implementation of the Hopfield model.^67^ The objective was to combine the parallelism and interconnectability of optics, which are linear phenomena, with bistable optical devices to provide the thresholding nonlinearity of the Hopfield model. The hardware proposed combined compound-semiconductor LEDs with photodiodes and electronics for an initial implementation of nonlinearity to be replaced by optical bistable devices in subsequent generations. While LEDs and laser diodes have become mature technologies, bistable optical devices have not.

The field of photonic neural systems has since experienced an immense diversification, with myriad efforts using free-space optics,^68^ fiber components,^69^ and on-chip integrated photonics.^70–72^ Along one branch of this tree, excitable lasers have been explored as spiking neurons.^69^ These lasers integrate several optical inputs, and release a laser pulse upon reaching threshold. These devices can be extremely fast, but consume too much power for scaling to the level of the human brain. It is also difficult to tailor the neuronal responses, as they are primarily determined by carrier and cavity dynamics, which are dictated by basic physics and not easily adjusted with circuit parameters. Excitable lasers can be used as spiking neurons in the broadcast-and-weight architecture,^73^ wherein each neuron is assigned a wavelength, and synaptic weights are established with microring resonators that attenuate the optical signals, much like waveglength-division-multiplexed fiber-optic networks. In conventional silicon photonics,^74^ such multiplexing employs around 10 channels. It may be possible to extend this to 100,^73,75^ but even this limited number of channels would require cumbersome control circuits to hold synaptic weights stable. The requirement of precise control at every synapse as well as the non-monotonic, rapidly varying Lorentizian line shape of microring resonances is not optimal for large-scale, unsupervised learning.

Phase change materials have also been explored for neuronal thresholding^76^ and as a means of implementing variable attenuation of photonic signals to establish a synaptic weight.^77^ This approach requires billions of photons to achieve synaptic weight modification. Relying on the properties of a material to achieve the complex computations occurring at a synapse limits functionality as compared to behaviors that can be tailored with integrated circuits.

Deep learning with continuous fields rather than spiking neurons is also receiving attention, and networks of on-chip, cascaded Mach–Zehnder interferometers are a prominent approach.^70^ Such networks excel at feed-forward processing operations, but are not conducive to the recurrent networks employed by spiking neural systems nor the activity-dependent plasticity necessary for unsupervised learning. The challenge arises because in meshes of interferometers, adjustment of one phase modifies multiple synaptic weights. While such a technique may be suitable for specific training algorithms employed in supervised learning,^78^ it appears cumbersome for unsupervised learning in large neural systems, where local activity at each synapse updates that synaptic weight.

Another exciting and related application space of photonics is in reservoir computing. This field has been innovative and productive in recent years.^79–83^ The objectives and hardware are only loosely related to the subject of large-scale cognition considered here, so further discussion is omitted.

As a broad point of contrast between the synapses discussed here and other systems using light for neural computing, most photonic neural systems encode information in the amplitude of optical signals received at a detector, and synaptic weights are established through modulation of the intensity of these optical signals. Whether phase modulation or direct amplitude modulation are leveraged, encoding synaptic weights in the intensity of light on a detector differs from the synaptic operations we are pursuing, where light is used for binary communication, and synaptic weights are established by electronic responses. This approach minimizes the optical power required and eliminates a source of noise. If synaptic weights are encoded in the intensity of an optical signal, noise from the light source is convoluted with the synaptic weight. With binary optical signaling the light level incident upon a synaptic detector does not influence the electronic response of the synapse, which is determined by the electronic circuits reading out the synaptic receiver. A binary response can be achieved with semiconductor receivers or superconducting circuits. In Ref. 50, we have shown that the response of a superconducting SPD is independent of the number of photons present in an incident pulse across four orders of magnitude of input intensity. Because no information is encoded in the light level, this form of optical communication does not suffer from typical shot noise. Provided one or more photons are received by the detector, a synapse event is communicated. The Poisson distribution gives the probability that zero photons are received. With an average number of five or greater photons transmitted per synapse event, the probability of receiving zero photons is less than 1%, a considerably lower error rate than biological synaptic transmission.^84^ We assume each neuronal light source will generate 10 photons per synaptic event to accommodate 3 dB of propagation loss while achieving 99% transmission success rate. All energy and power consumption estimates presented here use this value.

### C. Superconducting electronic neural systems

Many approaches to neural computing using superconducting circuits leverage the nonlinear properties of Josephson junctions. The objective of early superconducting neural circuits was to perform the weighted summation and thresholding operations required in the computational primitives of artificial neural networks.^85,86^ The circuits employed were similar to those utilized in superconducting digital logic, as were the basic concepts, such as using an up-down counter to implement synaptic weights.^86^ From the beginning, and continuing to the present,^87–89^ attention is paid to sculpting a sigmoidal transfer function to implement backpropagation as well as alternative circuits for achieving Hebbian-type learning.^85^

More recent efforts have broadened attention to consider also spiking neural systems, leveraging the inherent threshold and spike production of JJs.^90,91^ The most successful experimental effort to date demonstrated coupling of two neurons based on JJs, with inter-spike intervals on the order of tens of picoseconds.^92^ Additional progress has been made in synaptic memory technology based on magnetic JJs, wherein magnetic nanoclusters embedded in the tunneling barrier of a JJ are re-oriented by current pulses, providing a means to modify the junction critical current and dynamically reconfigure the response of a synaptic circuit.^91^ Such devices offer similar functionality to memristors being pursued for use in semiconductor-based neural systems.^93^

The superconducting circuits discussed in the present context have much in common with other contemporary efforts in JJ-based neural systems, particularly in the use of SQUIDs as the primary active element.^90,94^ One point of contrast is that our emphasis is toward high-capacity, analog flux-storage loops with diverse time constants as well as utilization of complex neurons with a dendritic tree for hierarchical information processing within each neuron, whereas other efforts are primarily focused on the high-speed^92^ and energy efficiency^88^ enabled by the use of superconducting circuits. This distinction is minute in comparison to the difference introduced by the choice to employ photonic communication. The challenge with using superconducting electronics alone to enable large-scale cognitive system is communication. In superconducting circuits, direct fan-out is usually limited to two, so for neurons to make thousands of connections, many stages of pulse splitters and active transmission lines must be employed. This leads to a cumbersome communication network requiring many JJs and severe challenges for wiring and routing. Reference 95 analyzed fan-out and fan-in in these systems and argued there is no fundamental limit to fan-out. Fan-in was identified as a limiting factor. However, the use of analog synaptic integration (SI) loops with high inductance eliminates the fan-in bottleneck.^36^

Fan-out challenges with superconducting circuits are not fundamental, and reasonable researchers in the field can disagree about the scale at which practical limits will be reached. For long-distance communication, pulses produced by JJs must be regenerated along active transmission lines. These transmission lines use JJs spaced periodically to re-transmit pulses, and the spacing of these JJs is set by inductance requirements. Using typical superconducting wires, a pitch of 100 *μ*m between these junctions is expected, meaning a neuron trying to reach a synapse on the other side of a 1 cm × 1 cm die will require 100 JJs for communication to that synapse. At the scale of a 300 mm wafer, 10 000 JJs would be required for long-range connections. Each of these JJs must be provided with a current bias. While many synaptic connections are local, long-distance connections are paramount, as described in Sec. II. Systems containing billions of neurons spread across hundreds or thousands of wafers, extending over meters, connected with active, superconducting transmission lines do not appear promising to me. Such a communication network may not be fundamentally impossible, but if the hardware for passive photonic communication proves feasible, scaling to massively interconnected spiking neural systems will be greatly facilitated. Most researchers pursuing superconducting electronics for neural computing are not seeking this scale of system.

### D. Optoelectronic neural systems

The superconducting optoelectronic approach to large cognitive systems described here utilizes similar superconducting circuits as Refs. 91, 92, and 96 for synaptic, dendritic, and neuronal computation, while leveraging light for communication, seeking scalability to massively interconnected systems. Optoelectronic integration may be most straightforward when combining superconducting circuits with silicon light sources operating at liquid helium temperature.

In addition to contrasting this approach to other existing work in the field, it is necessary to also consider what may seem a more straightforward route to optoelectronic intelligence. This route would involve spiking neurons based on waveguide-integrated light sources, as we have discussed, but instead of SPDs and JJs, semiconductor photodiodes and transistors would be employed. Pursuit of such hardware is impeded by the absence of light sources integrated with transistors. If there were a known means to integrate light sources as simple as transistors with silicon microelectronics, the landscape of computing would differ radically. Nevertheless, the proposition that superconducting electronics are more promising than photodiodes and MOSFETs for this application requires justification.

Our choice to focus on the superconducting approach is based primarily on three factors. First, superconducting SPDs dramatically reduce the brightness required of the light sources. While semiconducting detectors, such as avalanche photodiodes, can detect a single photon, the energy consumption negates the benefits of single-photon sensitivity in the system application under consideration. For scalable system integration, the semiconductor counterpart to a waveguide-integrated SPD working in conjunction with a JJ is a waveguide-integrated photodiode working in conjunction with a MOSFET. Such a semiconductor receiver is likely to require roughly 1000 photons to charge the capacitance of the MOSFET gate^97^ to initiate a synapse event. This factor of 1000 in photon power is matched by the factor of 1000 incurred to cool the superconducting system (see Secs. V and VI), so the net power consumption for light generation in semiconductor and superconductor systems is roughly equivalent. Yet the important distinction is that the superconducting system dissipates this power off chip in a cryocooler, whereas the semiconducting system requires the light sources to produce this power in the form of photons. Optoelectronic neural systems leveraging superconductors can make due with light sources providing 10 000 photons within a few tens of nanoseconds (30 nW continuous-wave equivalent), while a semiconducting counterpart will require light sources 1000 times brighter to attain the same firing rate. Achieving the former appears possible with inexpensive silicon light sources, while the latter is likely to require further advances in III-V sources. While exciting progress continues to be made in III-V integration on silicon,^98,99^ a central challenge remains to integrate these light sources intimately with electronics. The system under consideration requires fabrication of light sources by the millions across 300-mm wafers, which will surely be more cost effective if silicon devices as simple as transistors can be employed for light emission,^22^ a possibility that appears more likely with superconducting detectors and low-temperature operation.

The second factor driving our group to pursue the superconducting approach relates to multi-planar wafer-scale integration. Whether semiconductors or superconductors are used, artificial synaptic, dendritic, and neuronal circuits are not small. To accommodate millions of neurons and their synapses on a 300-mm wafer, on the order of 20 planes of photonic waveguides are required for communication, and a similar number of planes of electronic circuits are likely to be advantageous. For each plane of MOSFETs, high-temperature annealing steps are required for dopant activation, leading to processing challenges when integrating with metal wires, photonic waveguides, and light sources. This processing challenge is one reason extension of MOSFET processes to multiple stacked planes of transistors with copper interconnects has been difficult. Power dissipation and heat removal also come into play but may be less consequential in the context of spiking neurons with sparse activity. Superconducting electronic circuits are processed near room temperature, and the prospect of integrating many planes of JJs, SPDs, and wave-guides appears to us to be less restrictive. Multiple planes of active SPDs^100^ and JJs^101,102^ have been demonstrated.

The third factor steering us toward superconducting electronics relates to memory and learning. For a cognitive system of the scale under consideration, synaptic weight modification must be unsupervised and will be most readily realized if the signals that induce learning functions are the same signals, with the same current, voltage, or light levels, used for computing within neurons, and sent to synapses for communication. With superconducting circuits, single-flux quanta are used for computing, and single photons are used for communication. It appears possible for these same signals to update synaptic weights and enable learning, primarily by adjusting current biases to JJs. A close functional analogy would be to modify the voltage on the gate of a MOSFET in an analog manner, and indeed, this has long been the ambition of floating-gate MOSFETs for synaptic memory.^103^ However, the voltages required to change the charge on the gate are much higher than typical voltages used for computation elsewhere within the circuit, making it difficult to implement unsupervised learning based only on the signals already present in the network. These persistent challenges with floating gates have led many to look elsewhere for suitable adaptive circuits.^104^ While any one of the emerging approaches may lead to the desired memory operations, it is our perspective that the path to systems with lifelong learning and a multitude of memory mechanisms appear less formidable with Josephson circuits.

Despite these arguments in favor of superconducting electronics, several valid counterpoints can be raised. The requisite silicon light sources remain to be proven. Massively multi-planar fabrication of superconducting optoelectronic wafers is an ambitious technological undertaking. For many readers, the requirement of cryogenic operation is the most disconcerting aspect of the project. Several comments are in order. Low-temperature operation eliminates such systems from consideration for applications that require low system power consumption, such as mobile devices. But for systems with a million neurons, existing cryogenic technologies drawing a kilowatt of wall power are suitable, comparable to a home air conditioner in power consumption and complexity, but with cooling based on the thermodynamic properties of liquid helium. For larger applications, cryogenic operation may prove an insurmountable obstacle, although the scale of cryogenics used in superconducting magnets for particle colliders offers hope. The field of quantum information also provides an insightful lesson. Many types of qubits require operation at tens of millikelvin, necessitating the extra expense and complexity of dilution refrigerators. The environment at 4 K is comparatively balmy, and the required cryogenics are simpler and less expensive. Quantum information presently enjoys tremendous investment because these systems promise functions not otherwise possible. The same must be true of optoelectronic intelligence if it is to have a future. Anything that can be done with CMOS will be done with CMOS. If SOENs cannot achieve AGI that is otherwise unattainable, they will not be brought into existence. If they can attain unmatched cognition, someone is likely to be willing to pay for them, unless the expense is astronomical. The perspective presented here is that exactly this will come to pass: superconducting optoelectronic hardware will enable AI that simply cannot be achieved through other physical means. Low-temperature operation will be justified by the performance.

The vast majority of the universe is in thermal equilibrium with the cosmic microwave background at 2.7 K, below the proposed operating temperature of SOENs. In such a setting, all system power consumption estimates are reduced by a factor of 1000 from the numbers presented here, and the energy consumption per synaptic operation rivals that of the human brain, while enabling firing rates orders of magnitude faster. We should not expect technological intelligence to share our disposition to an environment where water is liquid; they may prefer to reside in an environment where helium condenses. If our goal is to answer scientific questions regarding the physical limits of cognition, low-temperature operation is not a fundamental impediment.

## V. SCALING AN OPTOELECTRONIC SYSTEM

The physics of light is complementary to that of electrons. Photons can co-propagate on a waveguide independently without capacitance. Waveguides can fan out without a charging penalty due to wiring. This is not to say, photonic communication can address an arbitrarily large number of recipients without consequence. For each new recipient, the number of photons in a neuronal pulse must increase. As destinations get further away, more energy is dissipated to propagation loss. These realities notwithstanding, it is feasible for devices communicating with photons to make direct, independent connections to thousands of destinations, thereby eliminating the need for the shared communication infrastructure that is the primary impediment to achieving AGI with electrical interconnections.

Having made this claim, the burden is upon us to provide evidence of the feasibility of photonic communication in large-scale neural systems. The large wavelength of light relative to the size of electronic devices causes concern for the size of optoelectronic brain-scale networks. To build confidence for the feasibility of the endeavor, I sketch here a vision of how such an optoelectronic neural system may be constructed. At the foundation of this vision is the assumption that the technology will utilize the fabrication infrastructure of silicon electronics and photonics in conjunction with fiber optics for longer-range communication.

**FIG. 6.**
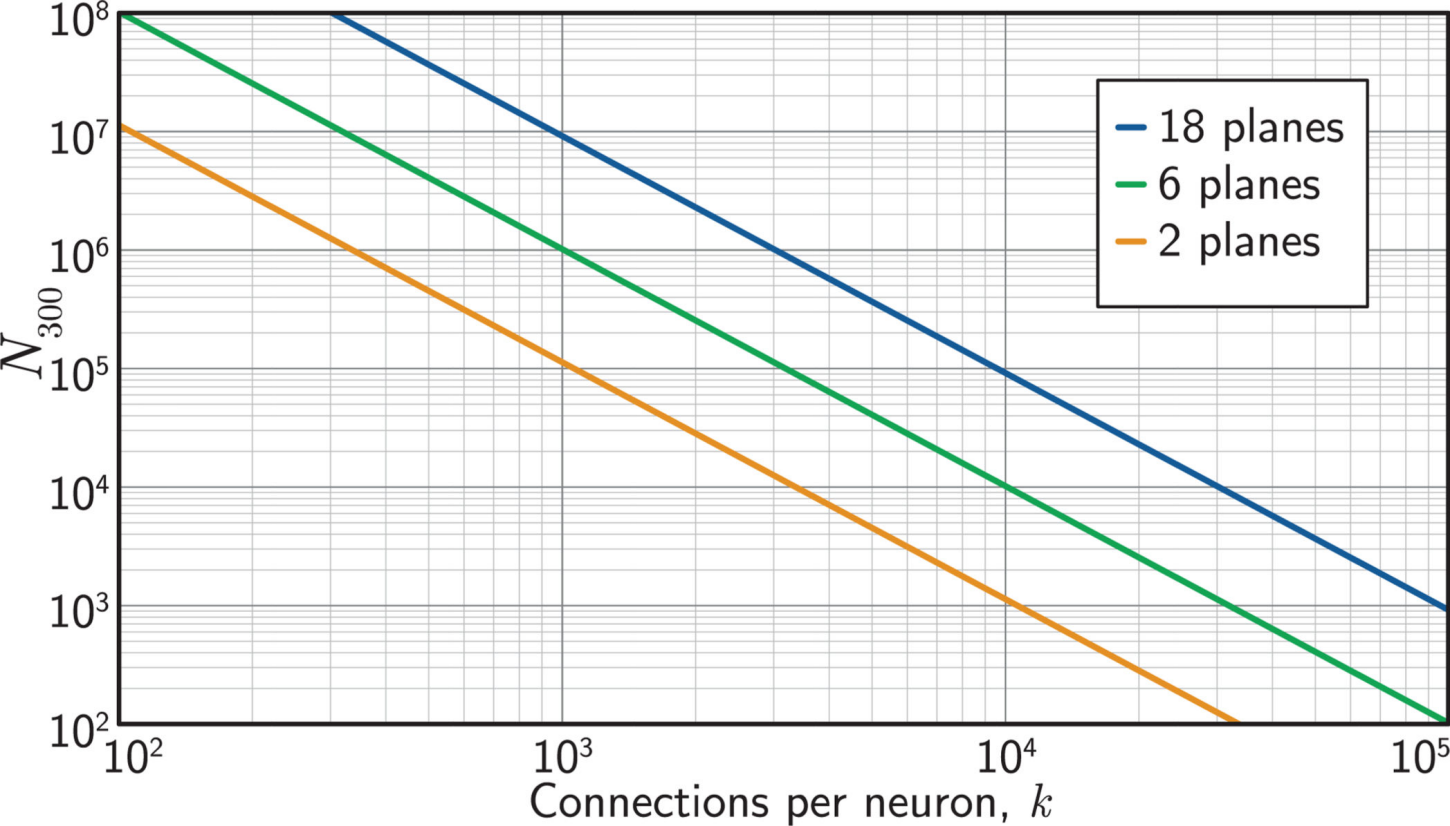
The total number of nodes that can fit on a 300 mm wafer (*N*_300_) as a function of the number of connections per node (*k*) for various numbers of waveguide planes in the wire-limited regime.^105^

**FIG. 7.**
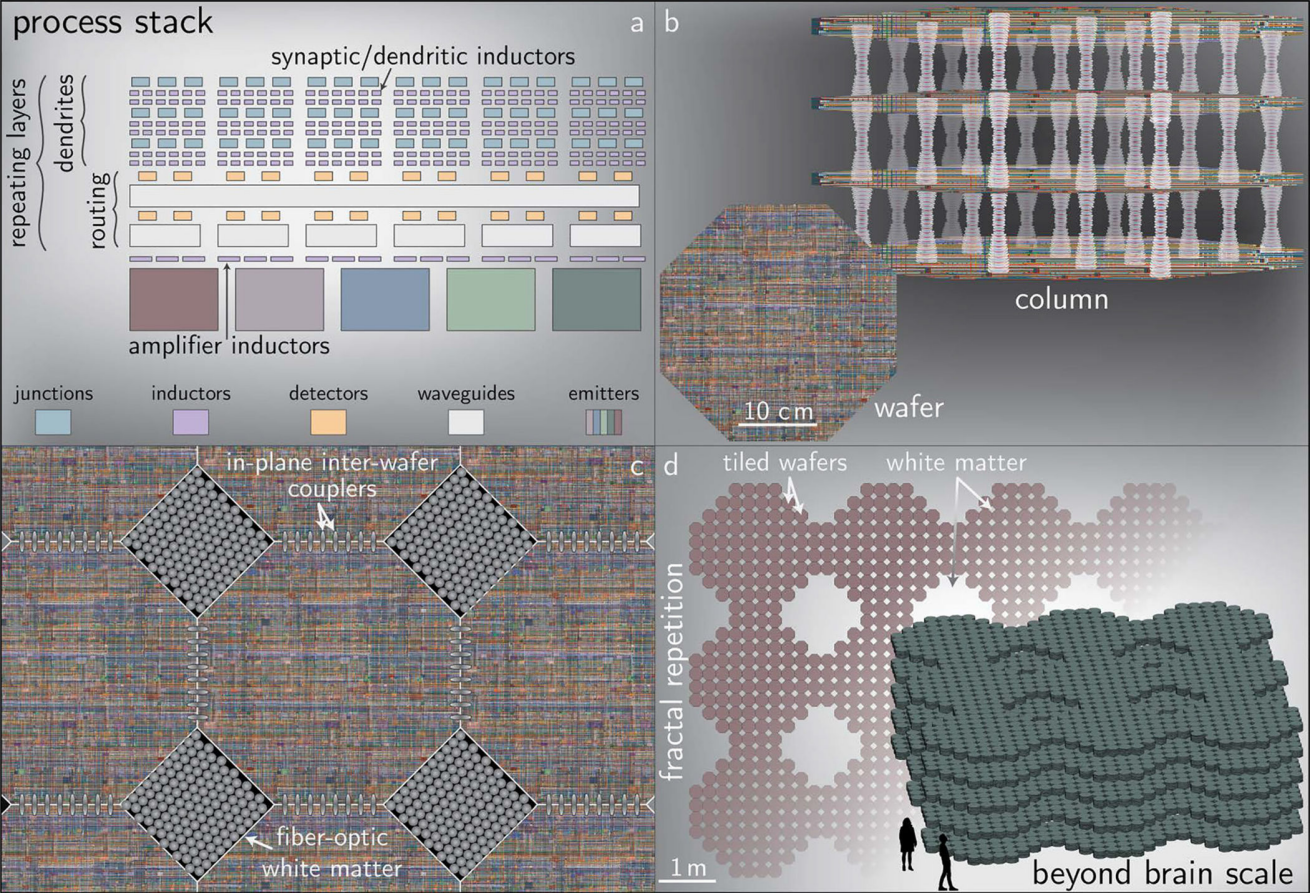
Hierarchical construction of optoelectronic neural systems. (a) Schematic of the process stack, with silicon light sources on a silicon-on-insulator wafer, waveguides and detectors above, followed by the Josephson infrastructure and mutual inductors for dendritic processing. (b) Vertical photonic communication between two stacked wafers. Liquid helium flows between the wafers of a column for cooling, and free-space links propagate without loss through the helium. The inset shows a schematic of a single 300 mm wafer, with neurons and routing, cut into an octagon for tiling. (c) Illustration of in-plane tiling. Lateral wafer-edge links connect wafers in a plane, and fiber optic bundles fill the voids between wafers for long-range communication. (d) A large neural system with multiple large modules, each containing hundred to thousands of wafers, enabled by photonic communication and the efficiency of superconducting detectors and electronics. Not shown is the fiber-optic white matter that would be woven through the voids between the octagons in this example hierarchical tiling.

At the wafer scale, light will be guided in multiple planes of dielectric waveguides^48,49^ [Fig. 7(a)], just as integrated electronics requires multiple wiring layers. To estimate the area of such photonic interconnection networks, we follow Keyes^105^ and approximate the number of neurons that can be supported on a 300-mm wafer by $N=2\sqrt{2}r^{2}{\left(p/wk_{in}\right)}^{2}$. Here, *p* is the number of planes of waveguides, *w* is the waveguide pitch (1.5 *μ*m), *k*_in_ is the number of waveguides entering the neuron, and *r* = 150 mm. The prefactor results from assuming octagonal tiling. This expression is plotted in Fig. 6. The estimate informs us that a 300-mm wafer with six waveguide planes can support roughly one million neurons if they each have one thousand connections. More involved analysis finds more planes may be needed.^36^ As a point of comparison to electrical neural systems, Ref. 106 finds that through multi-layer, wafer-scale integration of logic and memory, 250 × 10^6^ electrical neurons could fit on a 300 mm wafer. The trade-off is speed, as the shared communication network would limit the electrical neurons studied in Ref. 106 to 10 Hz operation when 1000 synaptic connections are made per neuron. Nevertheless, the message of Fig. 6 is that photonic routing results in large area consumption. An optoelectronic brain larger than that of a bumble bee will not fit on a single 300-mm wafer.

Optoelectronic intelligence will require communication between wafers. Wafers can be stacked vertically, and free-space optical links can send photons from a source on one wafer to a detector on a wafer above or below,^107^ as illustrated in Fig. 7(b). Assuming SPDs receiving vertical communication have a pitch of 25 *μ*m, a 300-mm octagon could support 10^8^ vertical communication links between two wafers. Considering wafers as laminar layers, as in cortex, such a configuration would result in roughly 5% inter-layer connectivity, similar to the fraction observed in mammals (Ref. 10, p. 286).

In addition to feed-forward and feedback free-space vertical coupling, lateral inter-wafer communication can be achieved at wafer edges, as shown in Fig. 7(c). In the tiling considered here, each wafer makes such connections to neighbors in the cardinal directions. With a 10 *μ*m pitch, 11 500 wafer-edge couplers could be supported in each direction. Such a system would demonstrate strong connectivity within the vertical stack of the wafers, and weaker lateral connectivity. The reader may recognize the columnar organization of the cerebral cortex.^5^

To achieve communication from within these columns to other regions of the network, optical fibers are ideal. Within the tiling under consideration, the square areas at diagonals between wafers can support fiber-optic bundles [Fig. 7(c)]. These optical fiber tracts are analogous to white matter in the brain. One such region could house a million single-mode fibers of 125 *μ*m diameter. These fibers will emanate from all wafers within the column, and if six wafers are stacked in a column, each wafer would have 167 000 output fibers to carry information to other regions. With one million neurons on a wafer, not every neuron would have access to a fiber for long-distance communication (unless wavelength multiplexing is employed). This again is consistent with brain organization, wherein the number of long-distance axons emanating from a region is smaller than the number of neurons within the region. Each of these fibers can branch as it extends through the white matter, so a neuron with access to a single wafer-edge fiber can establish multiple long-range connections. Recent progress in low-loss fiber-to-waveguide coupling^108^ indicates a potential future direction for such integration of fibers with on-chip waveguides, but significant advances in manufacturing are required to realize the coupling of dense fiber bundles to 300-mm wafers.

With this columnar configuration in mind, one can assess the feasibility of constructing a system on the scale of the human cerebral cortex (10^10^ neurons, each with thousands of synaptic connections). If a wafer holds a million neurons, a cortex-scale assembly requires 10 000 wafers. Assuming the volume of white matter scales as the volume of gray matter to the 4/3 power,^109^ the cortex-scale system would fit in a volume two meters on a side. While optoelectronic neurons are significantly bigger than their biological counterparts, it is not the absolute size that limits system performance. The relevant quantity for assessing scaling limitations is the ratio of the velocity of communication to the size of a neuron.^36^ Communication at the highest velocity in the universe more than compensates the large device size.

Regarding power, a single 300-mm wafer with a million neurons would dissipate one watt if the light production efficiency were *η* = 10^−4^, a conservative estimate. For the cortex-scale system of 10 000 wafers, the device power consumption with *η* = 10^−4^ would be 10 kW. A further cooling-power penalty of one thousand would be incurred if the system were operated in a background of 300 K. Thus, even in a conservative case of poor light production efficiency, an AGI on the scale of the human brain would consume 10 MW, the same order as a modern supercomputer. We are considering a system with roughly the same number of neurons and synapses as the human cerebral cortex, but with activity at 30 000 times the speed. While there is high uncertainty associated with scaling estimates of such an immature technology, these calculations indicate that artificial brain-scale systems with photonic communication and electronic computation may be feasible, a possibility with profound implications for the future of science and technology.

## VI. SUMMARY AND DISCUSSION

I have argued that artificial neural hardware should be designed and constructed to leverage photonic communication while performing synaptic, dendritic, and neuronal functions with electronic devices. Superconducting optoelectronic circuits elegantly implement these functions, in part because of the utility of Josephson nonlinearities for neural computation, and also because superconducting detectors enable few-photon signals, approaching the lowest possible energy for optical communication. We have demonstrated all of the core components and are working toward complete integration.

The approach to optoelectronic hardware described here is not without limits, and different factors limit performance at different scales. Regarding speed, the synaptic response is limited by the reset time of the SPD, which is between 10 ns and 50 ns depending on the material used. A response time of 50 ns limits the maximum neuronal firing frequency of the neuron to 20 MHz. For the silicon light sources we have primarily been pursuing, the emitter lifetime is on the order of 40 ns,^51^ giving a maximum firing frequency comparable to the 20 MHz figure determined by the speed of SPDs. In biological neural systems, conduction delays are an important factor limiting speed. Using light for communication greatly alleviates this concern, yet there does exist a scale at which the speed of light becomes the limiting factor. Within the 50 ns reset time of the SPD or the comparable 40 ns lifetime of the silicon emitters, light can travel 10 m in fiber. A system of this linear extent would contain at least an order of magnitude more neurons than an entire human brain. The scale set by this speed limit does not represent the maximum possible scale of an optoelectronic neural system, but rather the maximum possible volume of neurons that can communicate within the highest frequency oscillations of the system.

Regarding power consumption, cryogenic cooling plays a key role. The power required for cooling contains two contributions: the base-level power required to keep the environment below the superconducting transition temperature, even when the devices are inactive, and the additional cooling power required to remove excess heat generated by the activity of the circuits. The first factor is a few hundred watts for small systems, while the second factor is typically about one kilowatt of extra cooling power per watt of power dissipated by the devices. For small systems comprising a few thousand neurons each with a few hundred synapses on a 1 cm × 1 cm die, the devices will dissipate around a milliwatt,^36^ so the first factor dwarfs the second. The second factor does match the first until intermediate-scale systems with tens to hundreds of interconnected wafers, each dissipating 1 W when active. It is somewhere between the scale of a few thousand neurons on a die and a few million neurons interconnected across several wafers that we expect the performance of the system to exceed what can be accomplished without photonic communication and superconducting electronic computation. For large systems in excess of hundreds of interconnected wafers, the power dissipated by the active devices on the wafer and the associated cooling costs dominate. The power consumed by each wafer contains contributions from light sources, detectors at synapses, and JJs performing computations within dendrites and neurons. If light sources can be realized with 1% efficiency, each of these circuit components will contribute nearly equally to the total system power consumption.^43^

Despite these limits, this approach to AGI appears possible for physical and practical reasons. Physically, due to photonic signaling, it is possible to achieve efficient communication across the network for systems with orders of magnitude more than the 10 000 wafers comprising a brain-scale system. Reference 36 explores the communication-limited size of the system as a function of the frequency of network oscillations. Specialized processors with activity at 20 MHz (the gamma firing rate of loop neurons) can span an area 10 m on a side before delays limit communication. Modules with activity at 1 MHz (the frequency of corresponding theta oscillations in this system) could integrate information across an area the size of a data center within a single theta cycle.

On the practical side, fabrication of SOENs at industrial scale appears feasible. All the proposed circuits can be created on 300-mm wafers with existing infrastructure, such as a 45-nm CMOS node. Ten thousand wafers move through such a foundry every day. If dedicated to fabrication of optoelectronic intelligence, a foundry could produce multiple brain-scale systems per year. While the devices employed here depart from conventional silicon microelectronics, the same fabrication infrastructure can be employed.

What are the next steps to realize loop neurons and SOENs? Low-cost source-detector integration at the wafer scale is required. Demonstration of requisite plasticity functions would be an important milestone. Multi-planar integration of superconducting electronics would further build momentum. Active devices must be augmented with improvements in deposited dielectrics to enable many planes of routing waveguides with low loss. Hardware improvements will not lead to AGI without further theoretical insights. Conceptual advances are required to achieve high-performance neural systems, train them, and make them intelligent.

## Data Availability

The data that support the findings of this study are available from the corresponding author upon reasonable request.
